# BoNT-A for Post-Stroke Spasticity: Guidance on Unmet Clinical Needs from a Delphi Panel Approach

**DOI:** 10.3390/toxins13040236

**Published:** 2021-03-25

**Authors:** Alessio Baricich, Theodore Wein, Nicoletta Cinone, Michele Bertoni, Alessandro Picelli, Carmelo Chisari, Franco Molteni, Andrea Santamato

**Affiliations:** 1 Physical Medicine and Rehabilitation, Department of Health Sciences, Università del Piemonte Orientale, viale Piazza d’armi 1, 28100 Novara, Italy; alessio.baricich@med.uniupo.it; 2 Department of Neurology and Neurosurgery, McGill University, Montreal, QC H3A 0G4, Canada; twein@videotron.ca; 3 Department of Neurology and Neurosurgery, McGill University Health Center, Montreal, QC H4A 3J1, Canada; 4 Division of Neurology, Stroke Prevention Clinic, Montreal General Hospital, 1650 Cedar Avenue, Montreal, QC H3G 1A4, Canada; 5 Physical Medicine and Rehabilitation, Spasticity and Movement Disorder Unit, Policlinico Riuniti, University of Foggia, Viale Pinto 1, 71122 Foggia, Italy; andrea.santamato@unifg.it; 6 Physical Medicine and Rehabilitation, ASST Sette Laghi, 21100 Varese, Italy; michele.bertoni@asst-settelaghi.it; 7 Neuromotor and Cognitive Rehabilitation Study and Research Centre, Department of Neuroscience, Biomedicine, and Movement Sciences, University of Verona, 37134 Verona, Italy; alessandro.picelli@univr.it; 8 Unit of Neurorehabilitation, University Hospital of Pisa, 56126 Pisa, Italy; c.chisari@ao-pisa.toscana.it; 9 Villa Beretta Rehabilitation Center, Valduce Hospital, Via Nazario Sauro 17, 23845 Costa Masnaga, Italy; fmolteni@valduce.it

**Keywords:** stroke, spasticity, botulinum toxin, rehabilitation, Delphi

## Abstract

There is extensive literature supporting the efficacy of botulinum toxin (BoNT-A) for the treatment of post-stroke spasticity, however, there remain gaps in the routine management of patients with post-stroke spasticity. A panel of 21 Italian experts was selected to participate in this web-based survey Delphi process to provide guidance that can support clinicians in the decision-making process. There was a broad consensus among physicians that BoNT-A intervention should be administered as soon as the spasticity interferes with the patients’ clinical condition. Patients monitoring is needed over time, a follow-up of 4–6 weeks is considered necessary. Furthermore, physicians agreed that treatment should be offered irrespective of the duration of the spasticity. The Delphi consensus also stressed the importance of patient-centered goals in order to satisfy the clinical needs of the patient regardless of time of onset or duration of spasticity. The findings arising from this Delphi process provide insights into the unmet needs in managing post-stroke spasticity from the clinician’s perspective and provides guidance for physicians for the utilization of BoNT-A for the treatment of post-stroke spasticity in daily practice.

## 1. Introduction

Spasticity is a well-known disorder of motor dysfunction arising from upper motor neuron lesions due to stroke, spinal cord injury, multiple sclerosis, or traumatic brain injury [1]. The prevalence of post-stroke spasticity has been reported to range from 4% to 27% during the first 6 weeks following the initial event, 19% at 3 months, 21.7 to 42.6% at 4 and 6 months, and 17 to 38% at 12 months [2,3]. Patients with spasticity may suffer from decreased active movement, increased disability, and impaired function. Untreated, spasticity may lead to long-term secondary complications such as soft tissue contractures, decreased activities of daily living, pain, pressure sores, social isolation which all lead to decreased quality of life [4,5,6]. Botulinum toxin type A (BoNT-A) has been found to be effective and safe for treating focal post-stroke spasticity in the rehabilitation setting [7]. Several randomized and non-randomized controlled studies have documented the effectiveness of BoNT-A injections in reducing muscle tone in both the upper and lower limb in post-stroke spasticity [8,9,10,11,12]. According to the Royal College of Physicians guidelines, spasticity management and treatment with BoNT-A aims to relieve symptoms, improve function, and prevent deterioration [13]. Similarly, BoNT-A treatment has been shown to improve maladaptive positioning of the limbs [14], improve posture and gait [10,15,16], reduce predetermined disability parameters and pain [17], reduce caregiver burden [18], and improve person-centered goals [19,20]. 

Despite the considerable advances in the treatment of post-stroke spasticity with BoNT-A, a number of issues remain debated with unresolved clinical questions. Effective spasticity management is often challenging for clinicians in real clinical life since no evidence-based data are available to provide recommendations for: The optimal timing of intervention, what adjunctive therapies should be implemented, and what follow-up evaluations are required following injection of BoNT-A [21]. 

A recently published Italian survey focusing on spasticity management with BoNT-A addressed some of these debated issues, however, leaving some questions unsolved [22]. The authors revealed the existence of unmet clinical needs regarding the optimal timing of treatment, dosage selection, and the ideal time for follow-up. This survey of almost 80 clinicians specializing in spasticity had a unanimous consensus that spasticity should be identified as soon as possible following a stroke and that patients with changes in tone should be monitored closely. The survey also documented that most stroke patients’ have a 2–3 year delay to treatment following their stroke. Furthermore, clinicians are faced with the dilemma of how to effectively treat individuals with both upper and lower spasticity in a single session, given current dose limitations based on product monographs. To address these issues, a post-stroke checklist designed to identify treatable post-stroke problems, facilitate referral for treatment, and improve the standard of long-term management of stroke survivors was previously created [23]. A further Italian survey published in 2018 about the overall management of post-stroke spasticity with OnabotulinumtoxinA, also stressed the need for a major consensus on consistent clinical care models for the management of patients with post-stroke spasticity [24].

Based on these premises and awareness of the more clinical than methodological limits, it is clear that there is a need for a more practical and easier paradigm that can facilitate real-life settings.

Therefore, the objective of this project was to achieve a consensus within a group of expert Italian physicians regarding the most relevant unmet needs, as already highlighted in the previous survey, in order to depict Italian reality and propose interventions to optimize BoNT-A treatment in post-stroke spasticity. A Delphi survey design was used to integrate existing and well-supported evidence from the literature with the perspectives of experienced clinicians. 

The role of rehabilitation and adjunctive therapies will not be addressed in this project. The findings arising from the Delphi process will provide a framework that may be beneficial to worldwide clinicians in determining how to optimally manage patients with post-stroke spasticity when treating with BoNT-A. 

## 2. Results

The composition of the board committee and the panel of experts has been described in the Material and Methods. The board committee drafted initial open-ended questions regarding unmet needs in the following major areas, based on a previously published survey [22]: Timing of treatment, frequency of subsequent evaluations in stroke patients who have received or not received BoNT-A treatment, and BoNT-A dosage in relation to the severity of the patients’ post-stroke spasticity. The return rate was 100% for all three rounds. Of the statements achieving consensus, 11 were reached in the second round. The final statements and voting results are shown in the Appendix A (Table A1).

The expert panel agreed (83%) that, in order to prevent the negative consequences of spasticity, treatment with BoNT-A was needed as soon as post-stroke spasticity interferes with: Patients ADLs, medical team, caregiver or patient treatment goals. Almost all (91%) agreed that following an acute stroke, stroke survivors should be monitored for spasticity in need of treatment and that the duration of time from the initial stroke should not limit the evaluations. A consensus of 91% agreed that all stroke survivors should be followed for a minimum of one year. The Delphi panel also agreed (82%) that treatment with BoNT-A was indicated in patients where spasticity impeded patient function, ADL’s, medical team, patient and caregiver goals. Most of the participants agreed that, in order to optimize patient assessment, a follow-up evaluation was mandatory. A follow-up evaluation at 4–6 weeks after BoNT-A injection was considered the optimal time point by the majority (82%) of experts. There was also a high level of agreement (91%) that BoNT-A treatment and follow-up should be offered as needed, without any limitations due to the duration of post-stroke spasticity. 

A broad consensus (91%) was also achieved in establishing that treatment goals may remain the same or change from initial treatment to subsequent treatments depending on the evolution of the spasticity pattern. 

The expert panel reached a consensus (91%) on the need to modify treatment schemes and goal identification with each treatment cycle both in the initial evaluation and in subsequent injections over the long term. Experts agreed with a low grade of consensus (37%) that BoNT-A dose per muscle at first injection tends to be the same or lower than in subsequent injection over the long term. Lastly, they found no clear distinction between the upper limb (36%) and lower limb (9%) as the most commonly affected region treated with BoNT-A at first evaluation. 

## 3. Discussion

On the basis of a previously published Italian study, we decided to deeply analyze some consistent gaps found by the authors [22]. This study, published in 2017, was conducted, including 24 Italian neurorehabilitation units. Specifically, the survey focused on the use of OnabotulinumtoxinA and investigated BoNT-A treatment related to organizational structure theme, doses for upper and lower limb, and patient satisfaction or dissatisfaction. In parallel, unmet needs have also emerged. Hence, the present Delphi consensus’s main goal was to explore the existing and previously identified unmet needs in the overall management of post-stroke spasticity with BoNT-A, by focusing on the optimal timing of evaluations, how frequently to monitor stroke survivors and whether goals change over time. In our opinion, these results may help clinicians address common questions that arise in daily clinical practice when evaluating stroke survivors and in treating patients with post-stroke spasticity that may benefit from BoNT-A treatment. To the best of our knowledge, there are no Delphi-based studies providing insight into expert opinion on these issues. The previous surveys published in 2017 [22] and 2018 [24] provided a foundation to this further analysis, which is very specific and concerns well-defined topics: Initiation and follow-up, dosages, and treatment objectives. Although there is high-level evidence supporting the safety and effectiveness of BoNT-A in post-stroke spasticity (Grade A), there is little clinical trial-based evidence on how to manage, monitor, reassess, change goals when using BoNT-A for the management of post-stroke spasticity [25]. Based on the Delphi process, our results contribute to help optimize best clinical practice. Regarding the optimal timing to initiate BoNT-A, almost all respondents agreed that treatment is needed as soon as the spasticity interferes with the patient’s ADL’s or clinical goals as established by the medical team, patient, or caregiver. Treatment, evaluations, and intervention should not be influenced by the time interval from the initial stroke. According to the previously published literature, the optimal time for BoNT-A administration might be when spasticity becomes evident and bothersome to the patient, resulting in impairment of functions both actively and passively, causing disability or when it induces pain [26,27]. From our results, one of the aspects that certainly emerges is the onset of spasticity. Leaving aside the modalities for following patients after the acute event, which is affected by the different hospital situations, this aspect was accepted almost unanimously by all the participants.

Moreover, several recent studies have assessed the efficacy of early intervention with BoNT-A (i.e., <3 months post-stroke) [28,29]. As spastic hypertonia can develop during the first 12 weeks after stroke, early initiation of treatment may be beneficial on a symptomatic level. This could further reduce the development or progression of post-stroke spasticity and prevent the development of contractures and deformities [30]. Furthermore, there is increasing evidence that the effects of BoNT-A are not restricted to the neuromuscular junction but may also alter motor neuron central excitability at the spinal level. This includes increases in input resistance, decreases in rheobase currents for action potentials, and prolonged post-spike after-hyperpolarization in rats [31]. Studies in humans recognize that BoNT-A affects spinal synaptic transmission via its effect on cholinergic synapses of Renshaw cells in humans through retrograde transport [32].

Huynh et al. also demonstrated cortical excitability changes suggesting that clinical benefits of BoNT-A toxin relate to modulation of abnormal central reorganization (maladaptive plasticity) in post-stroke spasticity [33]. Early BoNT-A injection of the wrist and finger flexors in severely affected stroke patients within 4–6 weeks after stroke onset may prevent a disabling finger flexor stiffness six months later, which may reduce the development of contracture [29]. 

Based on these findings the expert panel also confirmed that monitoring patients, especially in the first year after stroke, is mandatory. Sandrini et al., in a study published in 2018, support that a post-stroke checklist may allow early detection of post-stroke spasticity, assist in the early management of disability and in rehabilitation planning [24]. Several predictors of early post-stroke spasticity have been proposed, including increased muscle tone, greater severity of paresis, hypoesthesia, and low Barthel Index score [3]. A more recent meta-analysis also identified age, motor, and sensory impairment based on a previously published SALGOT study [34] and hemorrhagic stroke as predictors for upper limb spasticity within the first month post-stroke [35]. Another critical issue that needs to be addressed is the long-term management of post-stroke spasticity with BoNT-A. Most of the previously published literature does not focus on this aspect of care, and the benefit of BoNT-A injections over time is not well documented in the literature. Therefore, according to the Delphi panel opinion, it was reported that BoNT-A was an effective therapy for decreasing post-stroke spasticity, and functional improvements can be achieved regardless of the duration of spasticity. It should be noted that long-term [36], repeated high doses [37], in both the upper and lower extremities of BoNT-A injections demonstrated a favorable safety profile with minimal adverse events, good patient satisfaction, and low incidence of immunogenicity [38]. 

In regards to visit intervals, the expert panel fully agreed that, due to the pharmacological activity of BoNT-A, a follow-up visit should be considered 4–6 weeks after BoNT-A injection, regardless of the different formulations. These findings are similar to the international guidelines for spasticity in adults treated with BoNT-A, as endorsed and issued by the Royal College of Physicians (UK) and by the European Consensus Table [14,39]. Follow up assessments are required to determine whether treatment goals have been achieved. However, in this regard, a consensus was not reached concerning whether there are differences in goals at first evaluation vs. goals selected in subsequent injections and long-term management. On the other hand, experts strongly agreed that BoNT-A treatment goals mostly depend on clinical scenarios as well as the patient, caregiver, and medical team goals and objectives, both at initial evaluation, first treatment, and in long-term management. Indeed, post-stroke spasticity management should be part of a goal-orientated program centered on the patient’s priority goals for treatment. Goal Attainment Scale (GAS) has been used in several studies evaluating outcomes after BoNT-A injections [20]. Pre-defined goals are ideally smart, achievable, and person-centered to optimize BoNT-A effects in areas of muscle tone, pain, active/passive functions, the burden of care, cosmesis, among others [40]. It was shown in the Upper Limb International Spasticity Study (ULIS)-II that 80% of patients with post-stroke upper limb spasticity treated with BoNT-A in a real-life clinical setting achieved their treatment goals [41]. Preliminary findings from the ULIS-III program identified pain as the second most common goal identified for treatment (25%), while goals of active function and range of movement have decreased when compared to ULIS-II [42]. These results are in line with a recent meta-analysis: Where BoNT-A was found to significantly reduce involuntary movements, spasticity-related pain, caregiver burden, as well as improve passive range of motion [16]. 

However, since individual goals for treatment may vary widely, the panel strongly agreed that treatment schemes should be re-evaluated continuously over time. Goals need to be reassessed continuously and compared to initial set goals, and the response to previous treatments needs to be taken into consideration for subsequent treatments and possible changing post-stroke spasticity patterns. In regard to the BoNT-A dose, the panel agreed that at the initial injection cycles, the dose per muscle tends to be the same or lower than in the long-term management phase. As previously stated, post-stroke spasticity leads to the development of structural changes in muscle over time by increasing intramuscular connective tissue and fat content, which may lead to a reduced response to BoNT-A injections [43]. Interestingly, in a previously published study, Picelli et al. showed that patients with higher spastic muscle echo intensity have a reduced response to BoNT-A injections, and a higher dosage of BoNT-A was required to treat these individuals [44]. 

Many aspects of care may influence the effectiveness of BoNT-A for the treatment of spasticity. Consequently, a systematic follow-up of treated patients is necessary in order to maximize the optimal treatment paradigm by taking into consideration, dosing, goal setting, and timing of evaluations. 

## 4. Conclusions

This Delphi survey focused on several critical aspects concerning the utilization of BoNT-A for the treatment of patients affected by spasticity by integrating data from International published guidelines, previous consensus statements, and published studies. The main limitation is related to the scope of the study. It would be interesting to undertake a similar study using the Delphi methodology with international experts and to observe and compare the results. The study’s main strength is related to the participants’ expertise, as most of them were expert physicians with extensive experience in spasticity management. Our results help worldwide clinicians to address unmet clinical needs in order to provide optimal spasticity management. Further randomized trials are needed to optimize BoNT-A utilization for the management of post-stroke spasticity.

## 5. Materials and Methods 

### 5.1. Study Design

A Delphi technique to obtain consensus on managing patients receiving BoNT-A therapy was used for this study. The name Delphi originates from Greek mythology. The Oracle of Delphi (Greece) was an omnipotent forecaster of the future and was “infallible in authority on all topics, spiritual, moral, ethical, or philosophical in nature.” The RAND Corporation developed the Delphi research method in the 1950s. They wanted to develop a research method using “expert opinion” from many rounds of consensus building, to predict trends and practices in the Cold War [45,46]. Since 100% agreement was seldom reached, the Delphi consensus methodology has the primary objective of identifying a central propensity in the group and providing the level of agreement reached [47]. The Delphi technique is an anonymous structured approach in which information is gathered from a group of participants to evaluate and comment on several items listed in a questionnaire: Based on the group response, participants then re-evaluate these items in subsequent Delphi rounds. The process is repeated until a consensus has been reached. An important advantage of the classic Delphi technique is anonymity, which can reduce the effects of dominant individuals or group pressure when using group-based processes to collect and analyze information. It is based on a series of questionnaires or “rounds” addressed to experts. The key features of this method are the anonymity of participants and controlled feedback [48,49]. A 3-round Delphi was proposed to generate pertinent items (round 1), explore preliminary consensus (round 2), and finally rate the short-listed items to determine priorities based on levels of consensus achieved (round 3) [50]. Three iterations are often sufficient to collect the needed information and to elicit consensus [51]. There was little consensus in the literature concerning the optimal sample size of a Delphi study. Some studies showed that experts who have similar training and general understanding in the field of interest allow for effective and reliable utilization of a small sample [52].

### 5.2. Scientific Committee and Expert Panel

The entire process of the Delphi, including the content of the statements and the recruitment of the participants, was supervised and validated by a scientific committee that was composed of 4 experts in spasticity management (Physical Medicine and Rehabilitation physicians) who were selected by the main medical societies in neurorehabilitation (Italian Society of Physical and Rehabilitation Medicine and Italian Society of Neurologic Rehabilitation). The scientific committee took part in identifying the study participants (expert panel) and assisted in drawing up, reviewing, and approving the specific questionnaire developed for use during the Delphi rounds. Twenty-one physicians from across Italy were invited to participate in this Delphi-based consensus survey. The criteria for their selection included professional knowledge and experience in the field of spasticity management. They were required to be authors or co-authors of papers dealing with “post-stroke spasticity and BoNT-A” published in peer-reviewed journals over the last 5 years. 

### 5.3. Data Analyses 

A database was created using SPSS Statistics version 20 by IBM (Armonk, NY, USA). Percentages for each response were calculated. A proportion within a range method was used to define consensus. Definition of consensus was established before data analyses. It was determined that consensus would be achieved if at least 75% of participants reached agreement or disagreement. This level of agreement has been considered appropriate in previous Delphi studies [53,54].

### 5.4. Delphi Panel: Round 1

During the 1st round (2 May 2019 to 13 May 2019), panel members received, via email, 12 open-ended questions and were asked to provide answers accompanied by essential support of literature (Table 1). Survey questions focused on the following domains: Treatment timing, monitoring, and patient-related issues depending on the objectives and onset of spasticity. A 2-week deadline was set for rating levels of agreement or disagreement with each statement. Based on the evaluations and feedback, a list of 29 possible statements was identified. 

### 5.5. Delphi Panel: Round 2

In the 2nd round, all the experts were requested to express their level of agreement/disagreement (a total agreement, partial agreement, disagreement). The items that did not achieve consensus (<75%) were dropped from the next round (Table 2). 

### 5.6. Delphi Panel: Round 3

In the 3rd round, 15 statements were finally achieved. As in rounds 1 and 2, a 2-week deadline was given for completion and return. At this stage, ratings obtained from the third round with 66% agreement were analyzed, and the board developed the final statements.

**Table 2:** Statements and consensus level at Round 2. TA Total Agreement; PA Partial agreement; DA Disagreement.

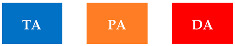	% TA	% PA	% DA
1. After the stroke onset, it is recommended to propose a treatment with botulinum toxin type A (BoNT-A) as soon as the spasticity interferes, and the clinical conditions require it, regardless of the time interval elapsed after the acute event (subacute phase)	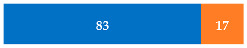		
2. It is necessary to monitor the patients after the acute event to verify the onset of post-stroke spasticity in order to propose a specific treatment for a period of at least one year	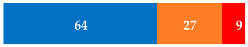		
3. It is necessary to monitor the patients over time after the acute event to verify the onset of post-stroke spasticity in order to propose a specific treatment, without a time limit but specifically in the first year	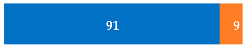		
4. After the onset of stroke, it is indicated to propose a spasticity treatment with BoNT-A in patients who are naïve in the presence of clinical needs, without time limitations	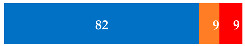		
5. For patients being treated with BoNT-A, a follow-up visit should be considered 4–6 weeks after BoNT-A injection	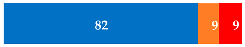		
6. For patients being treated with BoNT-A, after 12 months from the acute event a follow-up evaluation and a treatment are to be proposed every 3–6 months	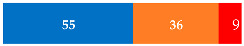		
7. For patients being treated with BoNT-A, after the acute event, it is advisable to continue with follow-up and subsequent treatments until it is necessary, without time limits	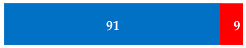		
8. The objective of treatment with BoNT-A, if the patient is in the sub-acute or chronic phase, may be the same or different depending on the clinical conditions and/or objectives; in the acute phase, there is a greater chance of positively interfering with phenomena of maladaptive plasticity	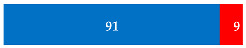		
9. The goals of spasticity treatment with BoNT-A do not change if the patient is in the sub-acute or chronic phase	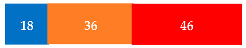		
10. The goals of spasticity treatment with BoNT-A are different if the patient is in the subacute or chronic phase	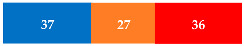		
11. For patients being treated with BoNT-A, both in subacute and chronic phases, the treatment schemes in place must be re-evaluated on each occasion in relation to the goals and in relation to the response to previous treatments	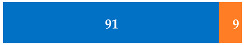		
12. The dose per muscle of BoNT-A used in the subacute phase tends to be the same or lower than in the chronic phase	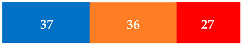		
13. The dose per muscle of BoNT-A used in the subacute phase tends to be lower than in the chronic phase	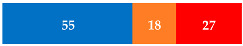		
14. In the subacute phase, treatment with BoNT-A is focused more frequently in the upper limb	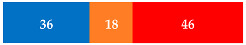		
15. In the subacute phase, treatment with BoNT-A is focused more frequently in the lower limb	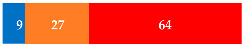		

## Figures and Tables

**Table 1:** The list of 12 open-ended.

1. How long after stroke onset is it indicated to propose treatment with botulinum toxin type A (BoNT-A)?
2. Until when should patients be monitored after the acute event to verify the onset of post-stroke spasticity in order to propose a specific treatment?
3. Until when, after the onset of stroke, is it indicated to propose treatment with BoNT-A in naïve patients?
4. For patients being treated with BoNT-A, how often should a follow-up evaluation and treatment be proposed for more than 12 months from the acute event?
5. For patients treated with BoNT-A, how long after the acute event is it appropriate to continue with follow up and subsequent treatments?
6. Is the goal of treatment with BoNT-A the same or different if the patient is in the subacute or chronic phase?
7. For patients treated with BoNT-A beyond 12 months from the acute event, how to evaluate the possibility of modifying the current treatment schemes (e.g., pattern to be treated, muscles to be treated, dose per muscle and overall dose)?
8. Are the dosages of BoNT-A, according to your experience, in the subacute or chronic phase the same or different?
9. Is the treatment with BoNT-A for the lower limb, in the subacute or chronic phase, also indicated in non-ambulatory patients?
10. Based on your experience, do you think there are differences in treatment frequency between upper limb and lower limb at an early stage?
11. Are there differences in the frequency of treatment between the upper limb and the lower limb in the chronic phase (more than 12 months after the acute event)?
12. Based on your experience, do you think there are differences in the frequency of multilevel treatment in the early phase and in the chronic phase (over 12 months)?

## Data Availability

The data presented in this study are available on request from the corresponding author.

## Abbreviations

***BoNT-A***	Botulinum toxin type A
***ADL***	Activities of daily living
***SALGOT***	Stroke Arm Longitudinal Study at University of Gothenburg
***GAS***	Goal Attainment Scale
***ULIS***	Upper Limb International Spasticity Study

## Appendix A

**Table A1:** Final statements from Delphi process survey.

Final Statements
1. After stroke onset, it is recommended to consider treatment with botulinum toxin type A (BoNT-A) as soon as spasticity interferes with patient, caregiver or health care team goals, regardless of the duration of time from the initial stroke.
2. It is necessary to routinely monitor stroke patients over time after the initial neurological event in order to verify the onset of post-stroke spasticity and initiate therapy when deemed necessary.
3. Routine evaluations for spasticity following a stroke should be done indefinitely.
4. Evaluation for spasticity should be for a minimum of at least one year from the initial stroke.
5. The duration of spasticity should not be a limiting factor for treatment with BoNT-A.
6. For patients treated with BoNT-A, a follow-up visit should be considered 4–6 weeks after BoNT-A injection.
7. For patients treated with BoNT-A, after 12 months from the acute event, follow-up evaluation and treatment should be proposed every 3–6 months.
8. Goal ascertainment with BoNT-A for post-stroke spasticity for patient at first inejction, may be the same or different on subsequent long term injections depending on the evolution of the spasticity patterns.
9. In the acute phase, there is a greater chance of BoNT-A therapy positively interfering with maladaptive plasticity phenomena.
10. For patients treated with long term BoNT-A, treatment schemes in place must be re-evaluated on each occasion to re-evaluate goals and ascertain the response to previous treatments.
11. Dosing BoNT-A for spastic muscles with similar severity at first injection tends to be lower or the same as in subsequent long term injections.
